# Manufacturing the Gas Diffusion Layer for PEM Fuel Cell Using a Novel 3D Printing Technique and Critical Assessment of the Challenges Encountered

**DOI:** 10.3390/ma10070796

**Published:** 2017-07-14

**Authors:** Arunkumar Jayakumar, Sarat Singamneni, Maximiano Ramos, Ahmed M Al-Jumaily, Sethu Sundar Pethaiah

**Affiliations:** 1 Mechanical Engineering Department, Auckland University of Technology, Auckland 1010, New Zealand; sarat.singamneni@aut.ac.nz (S.S.); maximiano.ramos@aut.ac.nz (M.R.); ahmed.aljumaily@aut.ac.nz (A.M.A.-J.); 2 Gashubin Engineering Pte. Ltd., 8 New Industrial Road, Singapore 536200, Singapore; sundar.energy@gmail.com

**Keywords:** PEM fuel cell, gas diffusion layer, membrane and electrode assembly, polyamide, titanium, carbon paper, selective laser sintering

## Abstract

The conventional gas diffusion layer (GDL) of polymer electrolyte membrane (PEM) fuel cells incorporates a carbon-based substrate, which suffers from electrochemical oxidation as well as mechanical degradation, resulting in reduced durability and performance. In addition, it involves a complex manufacturing process to produce it. The proposed technique aims to resolve both these issues by an advanced 3D printing technique, namely selective laser sintering (SLS). In the proposed work, polyamide (PA) is used as the base powder and titanium metal powder is added at an optimised level to enhance the electrical conductivity, thermal, and mechanical properties. The application of selective laser sintering to fabricate a robust gas diffusion substrate for PEM fuel cell applications is quite novel and is attempted here for the first time.

## 1. Introduction

Among the various fuel cell types, polymer electrolyte membrane (PEM) fuel cells are expected for future technology applications due to their versatile characteristics such as high power density (compatible for transportation), low operating temperature (60–90 °C), and dynamic response [1]. In addition, PEM fuel cells retain the best attributes of both batteries and internal combustion (IC) engines, making them a versatile energy conversion system [2].

**Figure 1:**
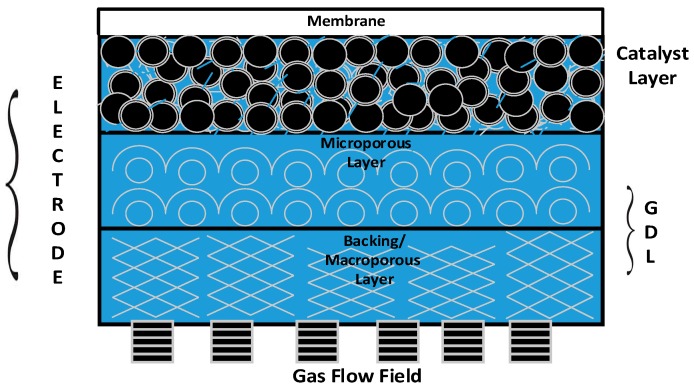
A 2D view of a gas diffusion electrode (GDE) indicating the catalyst layer and gas diffusion layer (GDL; comprising a backing layer and mesoporous layer, MPL).

The membrane and electrode assembly (MEA) is the prime component/heart of a PEM fuel cell stack, and consists of an electrolyte (proton-exchange membrane) sandwiched between two gas diffusion electrodes (GDEs). Figure 1 provides a 2D view of all the key functional components of a GDE. It is apparent from the figure that gas diffusion layers (GDLs) serve as an armour to protect the principal components—namely, the catalyst layer and membrane of the PEM fuel cell stack [3].

The desirable characteristics of a GDL include (i) relative stability in the fuel cell environment; (ii) good electrical conductivity; (iii) high permeability for gases and liquids; and (iv) elastic property under compression [4]. Carbon in the form of either paper or cloth are widely used GDL base materials, and both them have their own pros and cons [3]. Though the conventional carbon-based GDLs (non-woven carbon paper and carbon cloth) are functionally similar, they possess different structural characteristics, which might significantly influence the transport of heat, current, reactant gas, and water. Conventional GDLs are typically porous composites and comprise carbon-based material to enhance the electrical conductivity and polytetrafluoroethylene (PTFE) to improve hydrophobicity characteristics [5]. However, these materials contribute to the durability issues. Apart from the durability, the complex manufacturing process also augments the issues pertaining to PEM fuel cell commercialization.

## 2. GDL Degradation

The conventional GDL material suffers from several degradation issues, and the predominant ones are mechanical and electrochemical degradation. Mechanical degradation is due to the high compression and results in GDL deformation and changes in thickness due to the breakage and displacement of fibres under high pressures. Electrochemical degradation is due to the oxidation of carbon to carbon dioxide, and is illustrated in Equation (1):

*C + 2H*_2_*O → CO*_2_*+4H^+^ + 4e^−^, E*_o_ = 0.207 V vs. Standard Hydrogen Electrode (1)

However, operating a PEM fuel cell stack at such a low voltage (<0.207 V) is not practically possible. Incorporating a GDL that is free from carbon can be a promising solution to evade this issue.

**Figure 2:**
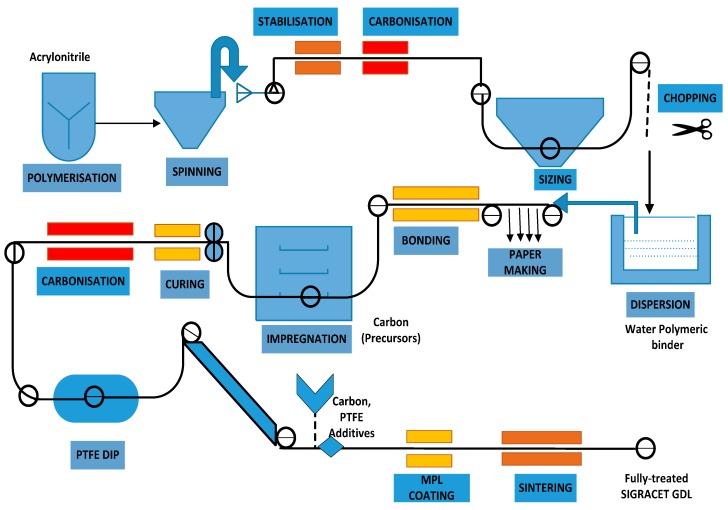
Manufacturing steps involved in conventional GDL fabrication (SGL 39 BC) [8]. PTFE: polytetrafluoroethylene.

Excess water accumulation (flooding) can accelerate the degradation of the catalyst and the gas diffusion layer due to polytetrafluoroethylene loss [6,7]. Apart from degradation issues, conventional GDL materials are fabricated by a multifaceted manufacturing process as shown in Figure 2, which is one of the prime reasons for its high cost.

The hypothesis of the present work is that the durability, complexity, and cost can be significantly improved in a positive manner by the 3D printing manufacturing technique. Therefore, the contribution to knowledge in the proposed work is to:

- Improve the durability of GDL as they are susceptible to electrochemical oxidation [9].
- Simplify the manufacturing process by an advanced 3D printing technique, which can be a promising solution to drastically reduce the costs and lead-time.

## 3. Experimental Procedure

The manufacturing process employed in the present work involves the use of a selective laser sintering (SLS) system, where the desired material in the powder form can be consolidated layer upon layer through laser heating. Sufficient inter-particle and inter-layer consolidation are achieved by optimising the laser energy flowing into the powder substrate [10]. The energy density (*E_D_*, J·mm^−2^) per unit area along the scan line can be evaluated as per Equation (2) [11]:

*E*_D_ = *P*/(*D* × *v*). (2)

The three critical parameters governing the SLS (3D printing) mechanism are laser power (*P*), scan speed (*v*) and beam diameter (*D*) [11]. The SLS system used for the work employs a beam deflection system (galvano mirrors) to achieve the laser scanning as required.

### Material Selection for SLS

Not all the materials that can be processed using the existing SLS infrastructure can be used to synthesize the gas diffusion material for the PEM fuel cell application. In the previous investigation, alumide [12] was used as the base material since it is stiffer than many other materials used in 3D printing and it contributes to good flexural strength and higher thermal load [13].

Though the mechanical and electrical characteristics of alumide are compatible for PEM operating conditions, one of the severe limitations from the fundamental chemistry perspective is that the aluminium present in the alumide is prone to oxidation in the PEM fuel cell environment, resulting in the possibility that metal ions formed could potentially damage the expensive membrane component. Other researchers [14,15,16] reported that aluminium bipolar plates exposed to a PEM fuel cell operating environment are prone to such a corrosion. Consequently, in the following work polyamide (PA) is used as the base material to develop the thin film samples.

The utilization of titanium structures has already been proposed by Hottinen et al. [17], and it is a safe material for GDL application. Titanium powder (US Research Nanomaterials, Houston, TX, USA) was added to polyamide in appropriate percentages to infuse appropriate conductivity, as polyamide is a non-conducting polymer, however well compatible with the SLS process.

In this present study, the base powder (PA) was sintered in a precise mode; appropriate functional material (Ti in the present study) was added to the base powder to attain the desirable functional characteristics. In the preliminary investigations, it was observed that sufficient electrical conductivity was not attained with 10% titanium, and consequently the experiment was performed with 20% and 30% titanium. For the 30% titanium composition, the laser was not able to sinter the composite powder, as the laser power oxidised the titanium metallic powder in the composite instead of selectively binding it. Thus, the composite with 30% titanium was too brittle and was left out of the subsequent investigation.

Based on this, the 20% Ti and 80% polyamide composite was considered for further evaluation. The composite powder was spread on the build platform layer upon layer, achieving a uniform dispersion and a flat top surface. The initial temperature of the powder bed was kept at around 70 degrees to keep the powder substrate dry and free of moisture. To study the diffusion and bonding of the titanium powder, the experiments were executed by varying the power as illustrated in Table 1 for a fixed scanning speed of 450 mm/s. These combinations of laser power and material composition were established based on trial and error experimentation and the resulting sintered structures. It may be noted that with increasing titanium content, the laser power required is decreased in order to achieve a continuous sintered layer. This is because the titanium component absorbs the heat from the laser and burns partly, which is an exothermic reaction, resulting in excessive thermal energies. Consequently, the higher the titanium content, the lower the energy density level for laser sintering.

## 4. Characterization

The characterization studies were performed to investigate the mechanical, physical, and electrical properties of the proposed gas diffusion material. The characterization studies involved in the present work are as follows:

(a) Surface and Elemental Energy Dispersive X-ray Analysis (EDX) characterization to investigate morphology and composition, Hydrophobicity, and Porosity measurement.
(b) Electrical characterization to investigate in-plane resistance.
(c) Thermal characterization to investigate thermal conductivity.
(d) Tensile characterization to investigate tensile strength.

The proposed paper considers SIGRACET*^®^* grade GDL 39 BC (325-μm thickness) as a baseline material. The unique characteristics of SGL 39 BC to consider is that it is denser and has a better water-retaining capability than its precursors (SGL 10 BC) [8]. Therefore, it is observed that SIGRACET^®^ grade GDL 39 will be an appropriate material for baseline consideration.

### 4.1. Surface Morphology—SEM

**Figure 3:**
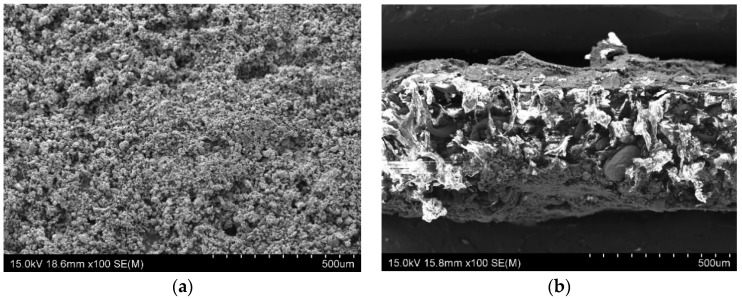
SEM image of the proposed gas diffusion material. (**a**) Surface; (**b**) Cross-section.

Surface characterization: The sample produced using SLS process was investigated and characterised using a Schottky field emission scanning electron microscope (SEM) (Hitachi SU-70, Tokyo, Japan). Figure 3 reveals the SEM image of (a) surface and (b) cross-section of the novel carbon-free gas diffusion material.

The Table 2 provides the elemental analysis of the proposed composite material.

### 4.2. Hydrophobicity

Contact angle measurement is a powerful diagnostic for understanding the interaction of GDL material with water. The degree of hydrophobicity is determined by the simple concept proposed by Zamora et al. [18], in which a 20-μL drop was deposited on a sample and after stand-up for 1 h, zoom shooting was conducted for the sample and the contact angle was measured between the droplet and the surface. The material exhibited predominantly hydrophilic nature with the contact angle measurement [θ ~ 20°].

### 4.3. Electrical Characterization

Electrical conductivity is a key property which directly influences the fuel cell performance [19]. Polymers filled with metal are of substantial interest because the electrical characteristics of such composites are close to metal properties with mechanical properties and processing procedures typical to that of plastics [20]. The figure of merit of the electrical characterization signifies the ease with which electrons transfer along the in-plane.

The electrical conductivity of the substrate was measured to be 1–10 S/cm; however, it was sensed to be low and consequently a nano-conductive platinum coating was provided by means of an ion sputter coater (Hitachi-E-1045, Tokyo, Japan) along both surfaces for few minutes, which surged the in-plane electrical conductivity of the substrate. The surface roughness of the substrate (as shown in the SEM image of Figure 3a) might be one of the factors that caused an effective physical absorption/diffusion of Pt towards the 3D matrix of the substrate; In addition, the grain sizes of Pt are smaller than gold, which validate the advantage of Pt over gold in ion sputtering. The four-wire Kelvin method was used to measure the electrical properties (in-plane resistance) of the GDL using the DMM 4040 meter (Tektronix, OR, USA).

### 4.4. Thermal Characterization

The thermal diffusivity of the sample material was measured using the laser flash apparatus LFA 467 *HyperFlash^®^* (NETZSCH, Bavaria, Germany). The sample was tested at several temperatures according to their behaviour in the desired temperature range of 25–160 °C. The measurements were carried out in a foil sample holder (Φ 25.4 mm) at the values of 25 °C, 80 °C, and 140 °C. In agreement with theory, the thermal diffusivity of the material decreased with higher temperatures, while specific heat values increased. Table 3 provides the thermophysical properties of polyamide-titanium composite.

### 4.5. Tensile Strength

Tensile test was performed using a TA.XT Plus texture analyser (Stable Micro Systems Ltd. Godalming, Surrey GU7 1YL, UK) to analyse the mechanical strength characteristics of the proposed material. ASTM D882 test method was piloted to estimate the tensile properties of the proposed thin films (as the thickness is less than 1.0 mm). To avoid tearing and premature specimen failure, the tensile test was conducted at a speed of 0.5 mm/s. The thin film material was clamped between two fixtures and tested to measure its tensile strength and was found to be approximately (ca.) 4 N/cm.

## 5. Polarization Curve

The MEA was fabricated as follows. Catalyst-coated membrane (CCM) was prepared by giving a coating of 0.5 mg Pt/cm^2^ on either side of the Nafion membrane. The fabricated GDL (by 3D printer) was attached on both sides of the CCM, after applying a thin coating of Pt black on the GDL side facing the membrane, such that the additional Pt loading was about 0.1 mg/cm^2^. The above MEA was placed in the fuel cell test fixture. The graphite plates with a serpentine flow channel were used for the single cell studies. The experiments were performed in both dry and humidified conditions (100% humidity at a cell temperature of 75 °C). The reactant gases—namely H_2_ and O_2_—were fed at a pressure of 15 psi. The cell was connected to a Hewlett Packard DC electronic load bank for the polarization studies. All the operating parameters were kept constant throughout the course of the experiment.

**Figure 4:**
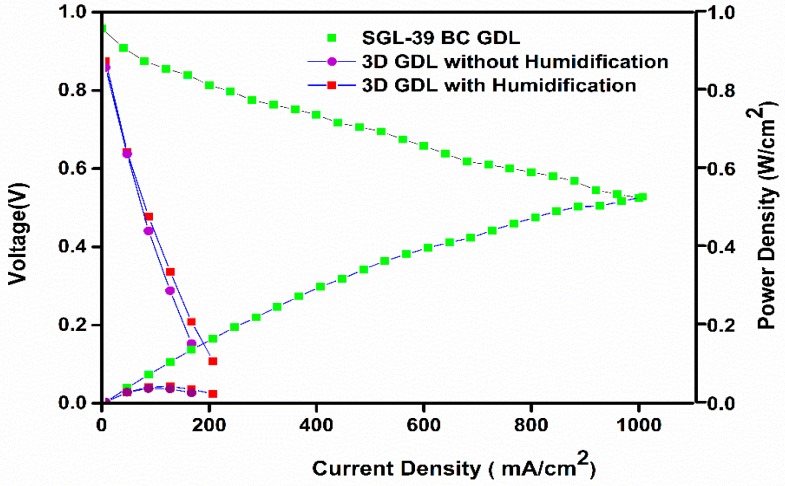
Polarization plots for the 3D-printed GDL [80% PA + 20% Ti] used membrane and electrode assembly (MEA) and normal MEA tested with humidified H_2_/O_2_ at 75 °C and 15 psi pressure.

Figure 4 displays the polarization curve, and it is obvious that the cell performance with 3D printed GDL showed a much more inferior performance than the commercial SGL-based GDL. However, it is inferred that the performance of the 3D printed GDL displayed a marginally improved performance with humidified conditions.

## 6. Discussion and Limitation

The values of the proposed material were compared against a wide range of conventional GDLs, and their values are illustrated in Table 4, which compares the properties of the proposed material (fabricated by 3D printing) and Carbon paper Sigracet™ 39 BC. The functional characteristics as specified in Table 4 authenticate that this material can be a hopeful candidate for GDL, as it is carbon free and possesses optimal multifunctional characteristics such as thickness, porosity, and conductivity. The low electrical conductivity is one of the prime limitations in this study, which might be attributed to the porous nature of 3D-printed GDL.

The characterization studies authenticate that the proposed material can be an economical alternative to the conventional carbon-based GDLs (woven carbon cloth and non-woven carbon paper) preparation route. One of the unique features of SLS to be compatible for GDL application is its porosity characteristics by default [21]. The numerical values of the various characteristics of the proposed GDL exhibited a minor deviation as elaborated in Table 4 due to the material’s anisotropic nature.

Though titanium has been previously used in the literature by Hottinen et al. [17], they were unable to achieve the desired thickness and porosity. However, in the proposed technique the thickness was around 430 µm, which is around 15% less than that made by Hottinen et al. The incorporation of the 3D printing technique is the prime factor to attain this level of fitness, where the complexity of the binder requirement is eliminated by the heat produced in the laser to bind the base powder to the metallic powder.

Though 20% titanium was the actual weight proportion used for the substrate preparation, it was sensed in the EDX (in Table 2) that weight as low as 14.8% actually contributed to the substrate formation. The following possibilities might have happened in the process:

- An increase in the Ti alone does not provide a feasible solution to enhance the conductivity, because with the present experimental set-up (non-inert atmospheric condition) the titanium present in the composite might be oxidised to titanium oxide (evident from the EDX of Table 2).
- Performing the experiment in argon conditions can drastically enhance the electrical conductivity, and under such an operating condition the increase in the Ti can actually enhance the electrical conductivity of the gas diffusion material and a higher percentage such as 30% or 40% can be feasible. In addition, the incorporation of ion sputtering to enhance the surface conductivity can be totally eliminated under that circumstance.

The authors assert that appropriate mixing of titanium particles with the base powder material using an advanced ball mill can also enhance the electrical conductivity, unlike in the present study where a manual mortar was used to mix the Ti powder to PA. Though in the proposed research the performance of the proposed GDL was much lower than the conventional GDL (SGL 39 BC), its manufacturing complexity is very simple and straight-forward. The DOE target of $5.5/m^2^ for a mass GDL production of 500,000 [22] can be very easily accomplished through this technique because the process is predominantly single-stage (as ion sputtering can be possibly eliminated in future), unlike multiple stages in the conventional route (evident from Figure 2), less residue is produced, and there is reduced lead time (improving the productivity) and complexity.

## 7. Conclusions

The principal implication of this work is that the carbon-based gas diffusion layer—which is the integral constituent in a PEM fuel cell stack—is prone to electrochemical degradation. To circumvent that, a novel additive manufacturing approach incorporating selective laser sintering was used to fabricate a non-carbon-based GDL directly from a 3D printing technique (additive manufacturing). This manufacturing route is an economical option, and has the substantial potential to achieve the Department of Energy ((DOE), USA target in the future by appropriately selecting the material and optimising the operating parameters. Though the performance is currently low, the authors ascertain that the fine-tuning of the SLS process parameters (such as SLS printer speed and laser power) and appropriate material selection and performing the experiment in the inert atmosphere can match its characteristics to be on-par with or superior to that of conventional GDL.

## Figures and Tables

**Table 1:** Laser power required to sinter various configurations of polyamide (PA)/titanium.

Laser Power (W)	Polyamide/Titanium Composition (%)
15	10/90
12	20/80
9	30/70

**Table 2:** The elemental analysis of the proposed composite material after selective laser sintering (SLS).

	C	N	O	Ti	Zr
S7-_PA	66.1	8.3	9.1	14.8	1.7

**Table 3:** Thermophysical properties of polyamide-titanium composite.

Temperature/°C	Thermal Diffusivity mm^2^/s	Specific Heat kJ/(kg·K)	Thermal Conductivity W/(m·K)
25	0.680	1.289	0.588
80	0.521	1.559	0.544
140	0.408	1.870	0.512

**Table 4:** Comparison of functional properties of the proposed material with Sigracet™ 39 BC [8].

Material Properties	Proposed Material (Polyamide-80% & Titanium-20%) Fabricated by SLS	Sigracet™ 39 BC
Thickness (µm)	430	325
Basic Weight (gm^−2^)	380	105
In-Plane Conductivity (S/cm)	1–10 * S/cm	170 **
Thermal Conductivity (W/(mK))	0.588–0.512 (Using Laser Flash Analysis)	0.25
Porosity (%)	ca. 42% (Using ImageJ)	52
Tensile Strength (N/cm)	≥4	NA

* Uncompressed; ** Compressed with 1 MPa.
